# The palisade cartilage tympanoplasty technique: a systematic review and meta-analysis

**DOI:** 10.1186/s40463-017-0225-z

**Published:** 2017-06-17

**Authors:** Caroline C. Jeffery, Cameron Shillington, Colin Andrews, Allan Ho

**Affiliations:** 1 Division of Otolaryngology-Head and Neck Surgery, University of Alberta, Hospital, 8440 112 Street, Edmonton, AB T6G 2B7 Canada; 2 Faculty of Medicine and Dentistry, University of Alberta, Hospital, 8440 112 Street, Edmonton, AB T6G 2B7 Canada

**Keywords:** Tympanoplasty, Palisade, Cartilage, Type I, Perforation, Tympanic membrane

## Abstract

**Background:**

Tympanoplasty is a common procedure performed by Otolaryngologists. Many types of autologous grafts have been used with variations of techniques with varying results. This is the first systematic review of the literature and meta-analysis with the aim to evaluate the effectiveness of one of the techniques which is gaining popularity, the palisade cartilage tympanoplasty. PubMed, EMBASE, and Cochrane databases were searched for "palisade", “cartilage”, “tympanoplasty”, "perforation" and their synonyms.

**Main body of abstract:**

In total, 199 articles reporting results of palisade cartilage tympanoplasty were identified. Five articles satisfied the following inclusion criteria: adult patients, minimum 6 months follow-up, hearing and surgical outcomes reported. Studies with patients undergoing combined mastoidectomy, ossicular chain reconstruction, and/or other middle ear surgery were excluded. Perforation closure, rate of complications, and post-operative pure-tone average change were extracted for pooled analysis. Study failure and complication proportions that were used to generate odds ratios were pooled. Fixed effects and random effects weightings were generated. The resulting pooled odds ratios are reported. Palisade cartilage tympanoplasty has an overall take rate of 96% at beyond 6 months and has similar odds of complications compared to temporalis fascia (OR 0.89, 95% CI 0.62, 1.30). The air-bone gap closure is statistically similar to reported results from temporalis fascia tympanoplasty.

**Conclusions:**

Cartilage palisade tympanoplasty offers excellent graft take rates and good postoperative hearing outcomes for perforations of various sizes and for both primary and revision cases. This technique has predictable, long-term results with low complication rates, similar to temporalis fascia tympanoplasty.

## Background

Tympanoplasty or tympanic membrane (TM) repair is one of the most commonly performed surgeries in Otolaryngology-Head and Neck surgery, with various types of graft and techniques advocated in the literature. The use of cartilage for tympanic membrane repair is well described [1–3] and has reported benefits of long-term graft survival, low recurrence and infection rates, and decreased development of tympanic membrane retraction pockets over time [3–5]. Authors have reported excellent functional results for small and large perforations [6–8] and often combine tympanoplasty with other middle ear procedures [2]. Cartilage tympanoplasty comprises a heterogeneous group of techniques including that the cartilage-perichondrium composite graft, diced cartilage, butterfly techniques, and palisade cartilage tympanoplasty [9–11].

Tos M. reviewed 23 different cartilage tympanoplasty methods and grouped them into six categories from A to F [10]. The palisade technique is considered a form of Group A cartilage tympanoplasty. The palisade technique specifically involves placement of 0.5 to 3-mm-thick pieces of cartilage placed side by side and often overlapping, under the TM remnant until the defect is covered [9]. This technique has been used with recurrent perforations, adhesive otitis media or tympanic membrane retractions and other mixed middle ear pathologies [12, 13]. Although several authors have reported success with this technique, we aimed to systematically review the literature on the use of cartilage palisades in Type 1 tympanoplasty and report clinical outcomes of this procedure including hearing and overall graft survival rate.

## Methods

### Search

A comprehensive search was undertaken using MEDLINE (from 1966), EMBASE (from 1980), CINAHL (from 1982), SCOPUS, and DissAbs in August, 2016. The keywords used were palisade, tympanoplasty, tympanic membrane, tympanic membrane perforation, ear drum, cartilage, and their synonyms. No limitation was placed on date or type of study.

### Inclusions/Exclusion

Abstracts of articles obtained from search strategies were independently reviewed by three authors CS, CJ and CA for further assessment. Strict inclusion and exclusion criteria were set *a priori*. English articles and non-English articles with accurate English translation were included. Studies were excluded if they included only pediatric cases, were case reports or reviews or the study design precluded the ability to extract palisade tympanoplasty data. Articles describing the clinical outcomes of palisade cartilage tympanoplasty were then reviewed in full and subjected to further inclusion and exclusion analysis. Studies describing palisade cartilage tympanoplasty performed in conjunction with other middle ear or mastoid surgery (e.g. concurrent ossicular chain reconstruction, mastoidectomy, etc.) were excluded. Specifically, only studies reporting hearing outcomes beyond 6 months were included. In addition to strict inclusion and exclusion criteria, the quality of studies were further assessed by grading their level of evidence based on the Oxford Centre for Evidence-Based Medicine Levels of Evidence for Therapy Studies [13].

### Data extraction

Data was then extracted from all articles, including patient demographics, study design, comparison groups, hearing outcomes, perforation closure rates and complications. Two authors extracted the data while a third verified the data extracted. Discrepancies were resolved by consensus. Specific outcomes of interest include graft success with at least 6 months follow-up, closure of the air-bone gap, and complications including middle ear infections, failure of graft survival, persistent perforation or otorrhea.

### Data synthesis and meta-analysis

Microsoft Excel (2016) was used to maintain extracted data and articles. Clinical outcomes from studies were pooled to determine rates of failure and complications in the cartilage palisade treatment group versus control. The overall take-rate represents with proportion of studies with complete closure of tympanic membrane perforation at 6 months or beyond. Study-specific odds ratios were calculated to obtain the odds of complications for both treatment and comparator groups. Both random effects and fixed effects models were applied to yield confidence intervals for pooled estimates of odds ratios.

## Results

**Fig. 1:**
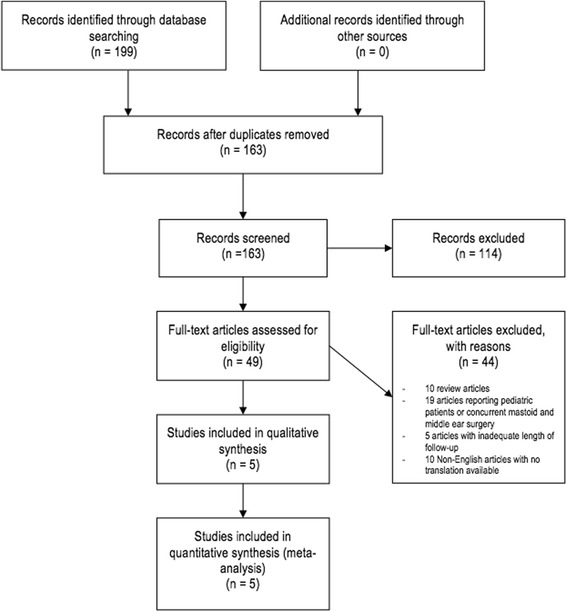
PRISMA Flow Diagram

The initial search yielded 199 articles, of which 163 were unique. Through screening of titles and abstracts, 114 were excluded based on initial criteria. The remaining 49 articles were reviewed then screened in detail by examining the full text and 44 more articles were excluded. Specifically, ten of the articles excluded were commentaries or reviews. Another nineteen articles were excluded due to their surgical method; some studies combined results for adult and pediatric patients or patients had concurrent middle ear (e.g. ossicular chain reconstruction) and mastoid surgery. Five more studies were excluded due to the lack of outcome measures or inadequate length of follow up. Ten more articles were excluded due to inability to obtain accurate English translations. This left 5 articles. Figure 1 is a flowchart of literature retrieved, application of inclusion and exclusion criteria, and resulting articles.

Of the included articles, 4 were retrospective studies and one was a prospective study. All articles compared their palisade group to a temporalis fascia group, and one also included patients undergoing repair with tragal perichondrium. Follow-up varied from 6 to 48 months. However, for analytical purposes, the results corresponding to the longest available follow-up time were used. See Table 1 for characteristics and level of evidence of included studies.

**Table 1:** Summary of included articles

Authors	Type of Article	Number of Patients Total (Palisade)	Comparators	Mean Age (Range)	Follow-up in months (Range)	Level of Evidence (Oxford Scale of Evidence)	Size of Defect
Khan et al. [14]	Retrospective cohort study	390(223)	Temporalis fascia	(11–57)	24 and 48 months	III	Both small and large
Kazikdas et al. [12]	Retrospective cohort study	51(23)	Temporalis fascia	27.6	Mean 18.7 months (7–33)	III	Subtotal perforations (perforation >50% of the whole TM)
Shishegar et al. [15]	Prospective cohort study	54(27)	Temporalis fascia	30	6 months	II	Subtotal perforations
Vashishth et al. [17]	Retrospective cohort study	90(30)	Temporalis fascia	24	12 months	III	Total/near total perforations excluded from fascia group, included in palisade
Demirpehlivan et al. [16]	Retrospective cohort study	120(19)	-Temporalis fascia -Tragal perichondrium	(15–64)	Minimum 12 months	III	Subtotal perforations

Khan et al. [14] included patients with both “small” and “large” perforations and is by far the largest study, with 390 total patients reported. Kazikdas et al. [12], Sishegar et al. [15] and Demirpehlivan et al. [16] only included subtotal perforations, with the defect being described as more than 50% of the area of the whole tympanic membrane. Vashishth et al. [17] selected patients for fascia or palisade group based on various risk factors. Specifically, the authors excluded patients with craniofacial abnormalities, revision tympanoplasties, near-total/total perforations, and persistently discharging ears from the fascia group. However, he did include these difficult to treat patients in the palisade group.

**Fig. 2:**
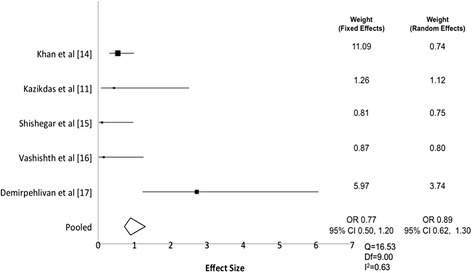
Forest Plot Demonstrating Pooled OR of complications comparing palisade tyampanoplasty to temporalis fascia

Individual study results, pooled graft-take rates and complication rates for palisade tympanoplasty versus comparator are found in Table 2. The pre-operative and post-operative air-bone gaps are reported in Table 3 along with average reduction in air-bone gap. The extracted proportion of patients experiencing complications in the palisade tympanoplasty and comparator groups were then used to generated odds ratios (OR). A weighted analysis of pooled ORs indicates no statistical difference in the odds of complications between palisade tympanoplasty and temporalis fascia tympanoplasty (i.e. confidence interval includes 1.0). Using a fixed effects model, which assumes fixed treatment effects, the overall odds ratio was 0.77 (95% CI 0.50, 1.20). Using a random effects model, which accounts for variability of treatment effects between studies, the overall odds ratio was statistically similar at 0.89 (95% CI 0.62, 1.30). Figure 2 demonstrates the forest plot for individual study OR estimates and the final overall estimate obtained by the two weighting methods.

**Table 2:** Individual study results, graft take rates and complication rates for cartilage palisade tympanoplasty compared to temporalis fascia

Authors	Palisade				Temporalis fascia			
	Number of Patients	Overall take rate	Complications	Type of Complications	Number of Patients	Overall take rate	Complications	Types of Complications
Khan et al. [14]	223	97.8%	10.0%	Persistent or recurrent perforation, otorrhea, infection	167	82.6%	17.3%	Persistent or recurrent perforation
Kazikdas et al. [12]	23	95.7%	8.7%	Perforation, otorrhea	28	75.0%	17.4%	Persistent or recurrent perforation
Shishegar et al. [15]	27	100.0%	4.3%	Infection, otorrhea	27	93.0%	25.0%	Persistent or recurrent perforation
Vashishth et al. [17]	30	90.0%	0.0%		60	83.3%	18%	Persistent or recurrent perforation, otorrhea, infection
Demirpehlivan et al.[16]	19	79.0%	10.0%	Persistent or recurrent perforation, infection	67	80.6%	16.7%	Persistent or recurrent perforation
Weighted average (SE)		96.0% (1.1%)	3.1% (1.0%)	Weighted average (SE)		82.5% (2.0%)	17.9% (2.1%)	

*SE* Standard Error

**Table 3:** Audiologic outcomes of included studies

Authors	Palisade			Temporalis fascia		
	Average Pre-operative ABG	Average Post-operative ABG	Reduction in ABG	Average Pre-operative ABG	Average Post-operative ABG	Reduction in ABG
Khan et al. [14]	30.7	7.1	23.6	32.9	8.1	24.9
Kazikdas et al. [12]	25.6	17.3	8.3	30.7	20.2	10.5
Shishegar et al. [15]	28.5	14.8	13.7	25.4	14.0	11.4
Vashishth et al. [17]	29.0	7.3	21.7	30.4	17.5	12.9
Demirpehlivan et al. [16]	28.0	15.0	13.0	24.5	14.0	10.5
Weighted average (SE)			20.9 (7.5)			17.9 (7.0)

## Discussion

The results of this systematic review and meta-analysis are clinically significant. Overall, the palisade cartilage tyampanoplasty technique has excellent functional results for Type 1 tympanoplasty with a 96% take rate at beyond 6 months. The air-bone gap closure is statistically similar to reported results from temporalis fascia tympanoplasty. Complications rates and long-term failure rates appear statistically and clinically comparable to tympanoplasty using temporalis fascia with no difference in the odds ratio of the two groups based on meta-analysis (0.89, 95% CI: 0.62 to 1.30). While this systematic review specifically excluded studies that reported results of palisade cartilage tympanoplasty combined with other procedures, an overall graft take rate of >97% has been reported in patients with who underwent combined palisade tympanoplasty with mastoidectomy for pathologies such as as cholesteatoma, adhesive otitis, and chronic mucosal disease [13, 18]. Our results are consistent with previous systematic reviews that demonstrate superior graft integration rate with cartilage tympanoplasty compared to temporalis fascia [8, 19]. Those studies did not examine palisade cartilage tympanoplasty alone by subgroup analysis, and thus, our systematic review and meta-analysis offers specific outcomes regarding the clinical effectiveness of this technique, the cartilage palisade tympanoplasty.

There are several limitations to this study. First, with the exception of the study by Khan et al. [14], which reported on 223 patients, the remaining studies had small cohorts of twenty three to thirty patients in the palisade group. These studies represented the results of single surgeons. In addition, there is likely significant publication and reporting bias of positive results. Since these studies are all non-randomized, selection bias remains an issue. In addition, the selection criteria used by authors for using the palisade technique was not consistent. While all the authors stated that operated ears must be dry and free of mucosal disease before surgery, the exact size criteria varied. Khan et al. included ears with “small” perforations, defined as less than 50% of the tympanic membrane and “large” perforations, defined greater than 50% [14]. In contrast, Cabra et al. selected patients with TM perforations >25% [3], while Kazikdas et al. included all primary tympanoplasties with >50% TM perforation [12]. Importantly, authors did not use digitally captured images prior to surgery to evaluate perforation size and there are inherent inaccuracies in the assessment and charting of perforations by subjective clinicians. Thus, future studies with large cohorts of patients need to accurately measure and report perforation size to enhance future comparability amongst surgeons and centers.

In addition to heterogeneity of perforation size, there is considerable heterogeneity in terms of patient age, co-morbidities, and other risk factors for graft failure. While the random effects model for pooled odds ratio attempts to address between study variance, we are unable to explain the drivers of heterogeneity or perform sub-group analysis due to the lack of patient-level data.

Finally, we specifically limited our review to studies of older children and adult patients. To our knowledge, only one study to date specifically examined the use of cartilage palisades in the pediatric population. Vashishth et al. examined outcomes of cartilage palisades over temporalis fascia at 6 months and 1 year in children and adult patients [17]. Although the authors demonstrated excellent results in the palisade group, we were unable to extract data for subgroup analysis (i.e. adult only or pediatric only) and thus excluded their paper from this review. However, given the paucity of literature and lack of consensus regarding pediatric tympanoplasty methods and outcomes, this represents an area in need of better research. Finally, the endoscopic approach to tympanoplasty is gaining popularity in Canada [20], but its adoption for the palisade tympanoplasty technique is unstudied.

## Conclusions

There is evidence that cartilage palisade tympanoplasty offers excellent graft take rates and good postoperative hearing outcomes for perforations of various sizes and for both primary and revision cases. This technique has predictable, long-term results with low complication rates, similar to temporalis fascia tympanoplasty.

## Abbreviations

OR: Odds ratio
TM: Tympanic membrane
